# Long noncoding RNA TMPO-AS1 accelerates glycolysis by regulating the miR-1270/PKM2 axis in colorectal cancer

**DOI:** 10.1186/s12885-024-11964-w

**Published:** 2024-02-21

**Authors:** Yingmin Jin, Aimin Jiang, Liying Sun, Yue Lu

**Affiliations:** Department of Gastroenterology, The First Affiliated Hospital of Harbin Medical University, 23 Youzheng Str, Harbin, 150001 People’s Republic of China

**Keywords:** Colorectal cancer, TMPO-AS1, miR-1270, PKM2, Glycolysis

## Abstract

**Background:**

Long noncoding RNA thymopoietin-antisense RNA 1 (TMPO-AS1) is recognized as a participant in cancer progression. Nevertheless, its biological function in colorectal cancer remains obscure and needs further elucidation.

**Methods and Results:**

First, we discovered enriched TMPO-AS1 in the tumor tissues that were related to poor prognosis. TMPO-AS1 knockdown enhanced SW480 cell apoptosis but inhibited invasion, proliferation, migration, and glucose metabolism. Further, MiR-1270 is directly bound with TMPO-AS1. MiR-1270 mimics were confirmed to inhibit cell proliferation, invasion, and glucose metabolism in our study. Mechanistically, miR-1270 directly is bound with the 3' untranslated regions (3'UTR) of PKM2 to downregulate PKM2. MiR-1270 inhibitors reversed the TMPO-AS1 knockdown’s effect on suppressing the tumor cell proliferation, invasion, and glycolysis, while the knockdown of PKM2 further inverted the function of miR-1270 inhibitors on the TMPO-AS1 knockdown.

**Conclusions:**

This study illustrated that TMPO-AS1 advanced the development and the glycolysis of colorectal cancer by modulating the miR-1270/PKM2 axis, which provided a new insight into the colorectal cancer therapeutic strategy.

**Supplementary Information:**

The online version contains supplementary material available at 10.1186/s12885-024-11964-w.

## Introduction

As the most prevalent gastrointestinal cancer, colorectal cancer has a continuously increasing incidence in young people with poor prognosis [1]. Many pathological and molecular therapies have been explored to serve as clinical interventions for colorectal cancer, which achieved much progress [2, 3]. However, the improvements in survival of colorectal cancer patients are still unsatisfactory [4].

Tumor cells often show intense abilities of proliferation, migration, invasion, anti-apoptosis, angiogenesis, and avoidance of growth inhibition. Recently, it has been found that tumors also can alter energy metabolism, which contributes a lot to helping the cells maintain the malignancy and making the glycolytic signaling pathway another essential feature of cancer [5]. Cancerous cells commonly display glycolytic pathway activation, which keeps the cells in high metabolic status and produces more lactic acid, resulting in decreasing extracellular pH [6]. High levels of glycolysis and low extracellular pH are associated with malignancy, including cancer cell proliferation and invasion, leading to a worse prognosis in colorectal cancer patients [7, 8].

Long non-coding RNA (lncRNA), with over 200 nucleotides in length, participates in many physiological and pathological processes, including immune response [9], growth and development [10], the incidence of diverse diseases, for instance, cardiovascular disease, neurodegenerative disease, and cancer [11]. LncRNA could modulate gene expression by working as microRNA sponges [12]. Lots of lncRNA play their roles in cancer genesis and development, such as HOTAIRs [13], MEG3 [14], MALAT-1 [15], H19 [16], and GCLNC1 [17]. Recently, thymopoietin-antisense RNA 1 (TMPO-AS1), functioning as a novel regulator to play roles in cancer, has drawn much attention [18, 19]. However, its roles in colorectal cancer haven't been clarified yet.

Here, we found that TMPO-AS1 regulated glycolysis in colorectal cancer by modulating miR-1270/PKM2 axis. This research illustrated the oncogenic effect of TMPO-AS1 and raised a new insight into the molecular mechanism of colorectal cancer.

## Materials and Methods

### Specimens

Totally 30 colorectal tumor tissues and the corresponding 30 adjacent normal tissues involved in the project collected from October 2022 to February 2023. were from the patients signing with informed consent at the First Affiliated Hospital of Harbin Medical University. All these patients haven’t received any chemotherapy or preoperative radiotherapy before. The tissue samples were kept in liquid nitrogen immediately after the surgical removal for subsequent detection. All experiments were permitted by the Ethics Committee.

### Cell culture and transfection

The colorectal cancer cell lines SW480 (Shanghai, Zhong Qiao Xin Zhou Biotechnology) were cultured in complete DMEM supplementary with 10% fetal bovine serum (FBS) and 1% penicillin/streptomycin in a 37℃-incubator supplement with 5% CO_2_. All the culture reagents, including the culture medium, penicillin/streptomycin, FBS, and trypsin, were purchased from Hyclone Laboratories, Inc. (UT, USA). For cell transfection, the lncRNA TMPO-AS1 siRNAs, and the relative control siRNAs were synthesized by Genscript Biotech (Nanjing, China). MiR-1270 mimics, miR-1270 inhibitors, and the relative control mimics and inhibitors were provided by Ribo Bio (Guangzhou, China). The sequence of si-TMPO-AS1 is 5’-GCU UGU CUG CAG GCA CUC AUA-3'; the si-control is 5’-GCG UUG CUG UCG CUC UAU UCU-3'. All siRNAs and miRNA mimics or inhibitors were performed transfection to cells for 48 h using Lipofectamine 3000 (Invitrogen, NY, USA).

### Cell viability assay

Applying the Cell Counting Kit-8 assay kit (CCK-8, Yeasen Bio, Shanghai, China), the SW480 cell proliferative rate was evaluated by detecting the change in cell viability. Seeded the cells in a 96-well plate first, and supplied CCK-8 solution 10 μL to each well simultaneously every day. After another 2 h of incubation, the absorbance with a microplate reader (Molecular Devices, CA, USA) at 450 nm.

### Flow cytometry

After transfection of siRNAs or miRNA mimics or inhibitors, SW480 cells were obtained. Rinse the cells twice by PBS, and dye them with FITC-AnnexinV and PI. Placed the mixtures for 15 min at room temperature in the dark, and then evaluated the cell apoptosis profile on the BD FACS Calibur (BD Bioscience, CA, USA).

### Wound-healing assay

After the transfection of siRNAs or miRNA mimics or inhibitors was indicated, the cells were made a scratch using the tips, and pictures of the scratch on the plate were taken. 48 h later, took pictures of the scratch on the plate again. Recorded the width of the scratch at 0 h and 48 h, and calculated the wound closure.

### Transwell assay

The cell invasion ability was evaluated via transwell assay. We carried out this experiment using the chambers containing 8.0-mm pore membranes (Millipore, Billerica, USA). We firstly used 200 μL of fetal bovine serum-free DMEM to re-suspend the SW480 cells and then plated all the cells as mentioned above into the top chamber pre-coated with a matrigel (Solarbio Life Sciences, Beijing, China). After that, we supplied a 0.5 mL DMEM containing 20% FBS to the bottom chamber as a chemoattractant for cells. 48 h later, the invaded cells were fixed for 30 min and stained with crystal violet solution for about 20 min. Next, we used the inverted microscope to take a photograph and count the quantity of the stained cells.

### Reverse transcription-quantitative PCR

Extracted total RNAs of SW480 cells by Trizol reagent (Invitrogen, NY, USA). After the extracted RNAs were quantified and purified, cDNA was obtained by transcribing 5 μg RNA. Following, qRT-PCR was implemented with SYBR qPCR Taq (Takara, Tokyo, Japan) under the procedure of 95 ℃ for 6 min, and 40 circles of the process of 95℃ for 60 s, 58℃ for 30 s, 72℃ for 60 s, followed with 72℃ for 10 min. Gene level was tested via the 2 ^−ΔΔCT^ method. The internal control refers to U6 and GAPDH. The primer sequences are as follows: TMPO-AS1-F: 5’-AGC CCA CAC ACT ACA GGC A-3'; TMPO-AS1-R: 5'- GCA CAA AAG CAG TAC GAC CT -3'; miR-1270-F: 5’-GGA GAT ATG GAA GAG CTG TGT-3'; miR-1270-R: 5’-CGA TCA GCA TTT CCA ATA TGC A-3'; PKM2-F: 5'- ACT GGC ATC ATC TGT ACC ATT G -3'; PKM2-R: 5'- AGC CAC ATT CAT TCC AGA CTT A -3'; U6-F: 5’-CGC AGC GGC AGC GGA TAT AC-3'; GAPDH-R: 5’-AAT GAA GGG GTC ATT GAT GG-3'; U6-R: 5’-ACG AAT TTG CGT GTC ATC CTT GCG-3'; GAPDH-F: 5’-GAG TCA ACG GAT TTG GTC GT-3'.

### Western blot

Extracted the proteins from SW480 cells with the radioimmunoprecipitation assay (RIPA) lysis and extraction buffer (Merk Life Sciences, Darmstadt, Germany) supplementary with phosphatase inhibitor and protease inhibitor (Sigma-Aldrich, Shanghai, China). The concentration of the extracted proteins was assessed using a commercialized Bradford protein assay kit (BOSTER Biotechnology, Wuhan, China). Protein of the same quantity (50 μg) was electrophoresly separated on 12% SDS-PAGE gel, and the protein in gels was transferred to methanol-pretreated PVDF membranes (Millipore, Darmstadt, Germany). The membranes with separated proteins on them were then submerged with 5% skim milk solution at 25℃ for 90 min, and immersed in indicated diluted primary antibodies at 4 °C for at least 12 h, followed by incubating with indicated secondary antibodies at the dilution of 1:5000 at room temperature for 90 min. In the present study, the primary antibodies against PCNA, Ki67, MMP2, and MMP9 were offered by Abcam (Cambridge, UK). Antibody against β-actin (Proteintech, Wuhan, China) was the internal control. Diluted all primary antibodies at the ratio of 1:1000. The protein bands were observed by the chemiluminescence in the enhanced ECL immunoblotting system (Tanon, Shanghai, China) and analyzed by ImageJ.

### Glucose uptake assay

Glucose Uptake Colorimetric Assay Kit (Biovision, CA, USA) was applied for the measurement of cell glucose uptake accordingly. The SW480 cells were inoculated in a 96-well dish (1500 cells per well). Before the detection, the cells were removed from the medium rinsed twice by 1 × PBS, and cultured in an FBS-free medium overnight. The other day, after the cells 3 times with 1 × PBS were washed, the cells were subjected to starvation from glucose for 40 min by the (Krebs–Ringer-Phosphate-HEPES) KRPH buffer. Then 10 μL 2- deoxyglucose (2-DG) was mixed into the reaction system for 20 min, followed by 1 h of enzyme mixture incubation. Added the extraction buffer and the Neutralizing buffer and then added the mixture of glutathione reductase and DTNB. Read the absorbance at 412 nm by using an automatic microplate reader (Molecular Devices, CA, USA).

### Lactate production assay

The Lactate Colorimetric Assay Kit (Elabscience, Wuhan, China) was utilized to measure the lactate produced by cells under the instruction of the manufacturer. Briefly, the cell culture supernatant (50 μL) was added to the microplate plate, then a 50 μL lactate enzyme mixture was mixed with the samples and kept at 25℃ for 25 min. Finally, read the absorbance at 450 nm by using the automatic microplate reader.

### ATP production assay

The ATP produced by cells was examined utilizing the commercialized ATP detection kit (Elabscience, Wuhan, China). Briefly, the cells using lysis buffer in the kit, performed 10 min of centrifugation at 11,000 g, and removed the supernatant. 50 μL samples were added to a 96-well dish, followed by an addition with a working solution at a volume of 100 μL. Next, the luminescence of the mixture was detected by an automatic microplate reader (BioTek, Winooski, VT, USA).

### Dual-luciferase reporter assay

Starbase was utilized for the prediction of the TMPO-AS1 binding sites of miR-1270 and the miR-1270 binding sites of PKM2. The pMIR-GLO report luciferase vector was obtained from Genepharma (Shanghai, China). The wild type (WT) of the seed sequence of miR-1270 in TMPO-AS1 or 3'UTR of PKM2 and the mutant type (Mut) of the seed sequence were magnified and implanted into the pMIR-GLO vector to construct wild type and mutant type luciferase plasmid. The wild type or the mutant type plasmids were co-transfected with the control mimic or miR-1270 mimic to SW480 cells for 48 h via Lipofectamine 3000 (Invitrogen, NY, USA). The luciferase signals were detected with a commercialized assay kit (Yeasen Biotechnology, Shanghai, China) and normalized to the Ranilla luciferase activity.

### Starbase database analysis

We applied Starbase v3.0 (http://starbase.sysu.edu.cn/) database to obtain the potential binding miRNA of TMPO-AS1. In this study, the binding of miRNA of TMPO-AS1 was predicted by Starbase v3.0.

### Statistical analysis

GraphPad Prism (7.0 version) was applied to perform the statistics in this project. Means ± Standard Deviation presented all data, and all trials were conducted 3 times. One-way ANOVA or Unpaired Student t-test was for testing the significant differences between pair groups, and Fisher's LSD test was for multiple groups. *P* < *0.05* means statistically significant.

## Results

### TMPO-AS1 is significantly enriched in colorectal tumor tissues.

**Fig. 1:**
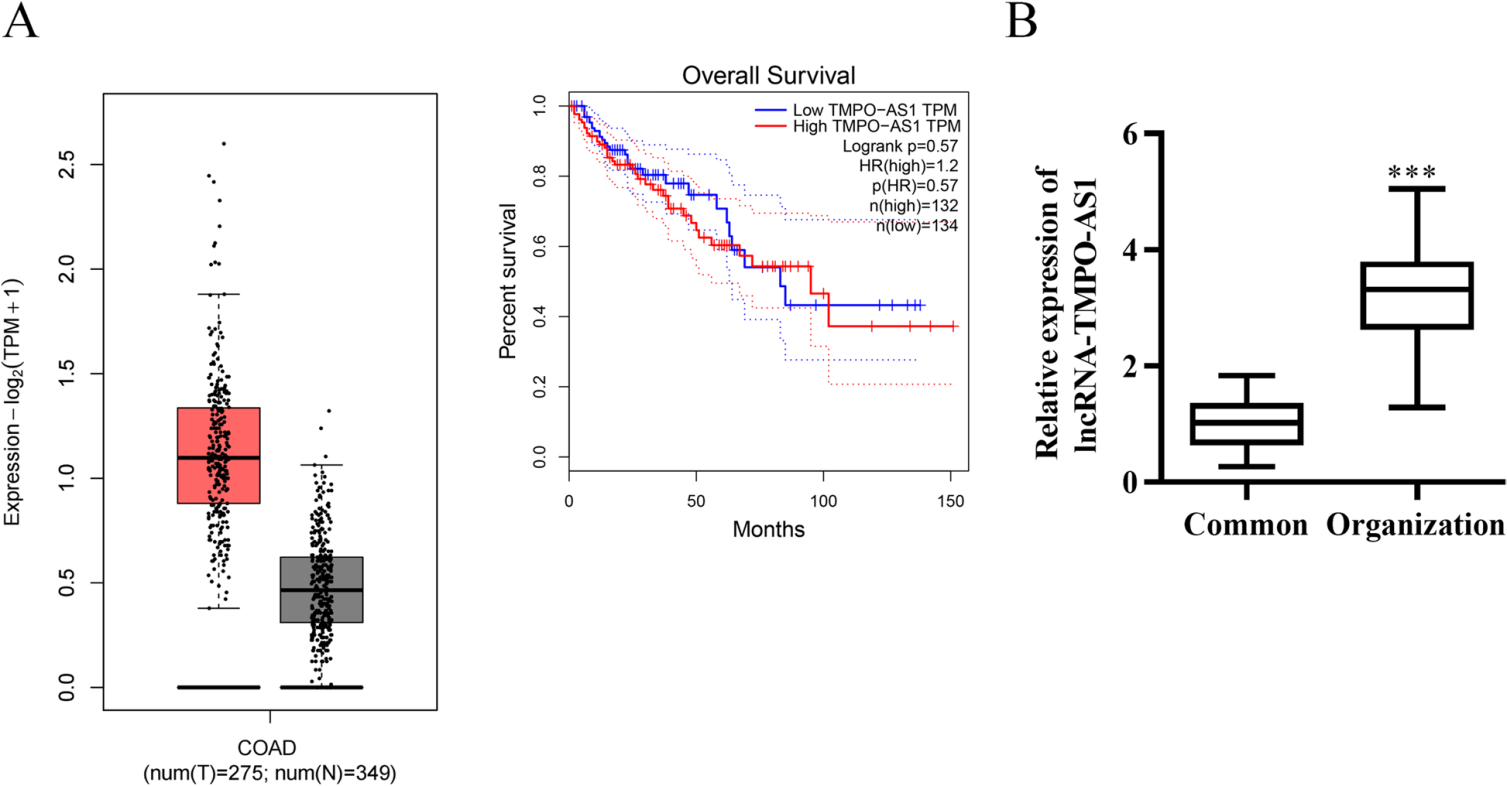
TMPO-AS1 is significantly enriched in colorectal tumor tissues. **A** GEPIA database showed TMPO-AS1’s expression profile in colorectal tumor (*n* = 275) and the normal tissues (*n* = 349), and the correlation between TMPO-AS1 abundance and the prognosis of the colorectal cancer patients. **B** qRT-PCR exhibited TMPO-AS1’s expression in the clinical surgery-derived tumor tissues and normal tissues. *P < *0.05*, **P < *0.01*, ***P < *0.001*, *vs*. Control group.

This study used the GEPIA database to obtain the TMPO-AS1 expression profile from a panel of 275 tumor specimens and 349 control specimens in the TCGA database. It was found that TMPO-AS1 showed a significant upregulation in the colorectal tumor tissues (Fig. 1A). Then we explored the relation of TMPO-AS1 expression with the disease prognosis by the GEPIA database. No significant difference was shown in the overall survival of patients between high- and low- low-expression groups (Fig. 1A). We further assessed TMPO-AS1 abundance in colorectal tumor tissues from clinical surgery. The results displayed that TMPO-AS1 abundance was markedly elevated in tumors than in normal adjacent tissues (Fig. 1B), consistent with the above database analysis in Fig. 1A. These results indicated that TMPO might have oncogenic effects on colorectal cancer.

### TMPO-AS1 knockdown restrains the malignant characteristics of colorectal tumor cells

**Fig. 2:**
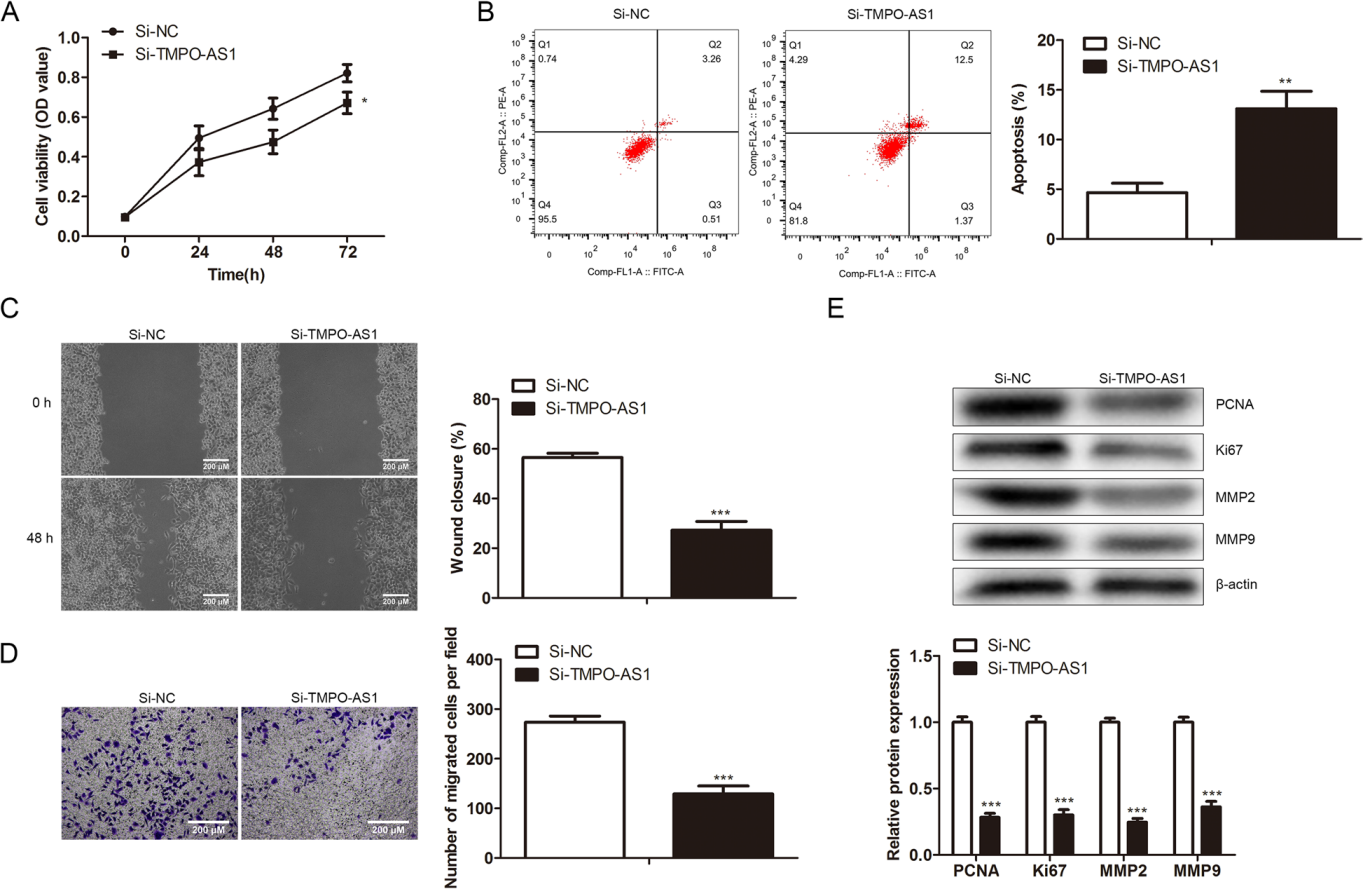
TMPO-AS1 RNA interference restrains the malignant characteristics of colorectal tumor cells. The SW480 cells were performed with 48 h of transfection of 40 pmol/mL TMPO-AS1 siRNAs and relative negative controls. **A** Cell viability and proliferation of SW480 cells in the indicated groups. **B** Flow cytometry assay was implemented to determine the SW480 cells' apoptosis. **C** Cell migration viability after transfection was assessed through wound-healing assay. **D** SW480 cell's invasion ability was revealed through transwell assay. **E** Levels of PCNA, Ki67, MMP2, and MMP9, and internal control β-actin was measured though western blot. *P < *0.05*, **P < *0.01*, ***P < *0.001*, *vs*. Control group.

The influence of si-TMPO-AS1 on tumor cell apoptosis, migration, proliferation, and invasion was studied to explore the TMPO-AS1 effect on colorectal cancer progress. We knocked down TMPO-AS1 in the SW480 cells. Cell viability and flow cytometry results displayed that TMPO-AS1 knockdown suppressed cell proliferation (Fig. 2A), but induced more cell apoptosis in the SW480 cells (Fig. 2B). In addition, TMPO-AS1 knocked down cells exhibited less migration ability and less invasion ability (Fig. 2C, D). We further detected the proliferation-related and migration-related protein expression in cells knocked down by TMPO-AS1. PCNA and Ki67 are common proliferative biomarkers used in cancer [20, 21]. Matrix metalloproteinases (MMPs) could degrade the extracellular matrix, which boosts the invasion, migration, and metastasis of tumor cells [22]. The data in Fig. 2E indicated downregulated levels of Ki67, PCNA, MMP2, and MMP9 in the TMPO-AS1-knockdown cells. All the results clarified that TMPO-AS1 knockdown restrained the malignant characteristics of colorectal tumor cells, supposing that TMPO-AS1 had oncogenic effects in colorectal cancer.

### TMPO-AS1 knockdown represses the glucose metabolism of the colorectal cancer cells

**Fig. 3:**
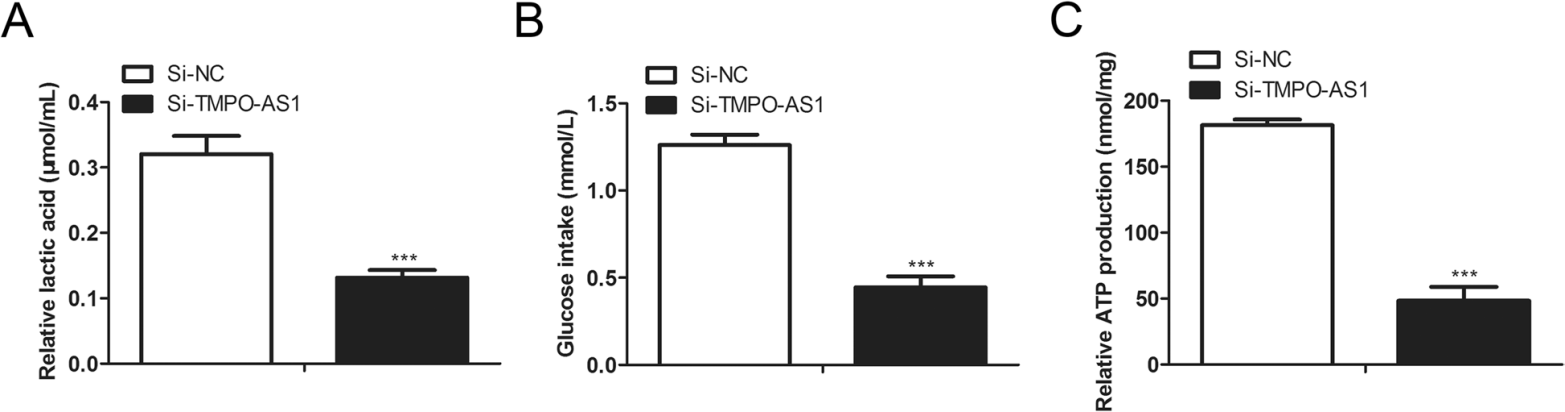
Knockdown of TMPO-AS1 represses the glucose metabolism of the colorectal cancer cells. The SW480 cells were performed with 48 h of transfection with 40 pmol/mL TMPO-AS1 siRNAs and the relative negative controls. **A**, **B**, **C** The lactate secretion, glucose uptake of SW480 and ATP production were evaluated by the indicated commercialized assay. *P < *0.05*, **P < *0.01*, ***P < *0.001*, *vs*. Control group.

Glucose metabolism acts as an essential part of the tumor process, and inhibition of glucose metabolism leads to tumor cell death [23, 24]. We then explored whether TMPO-AS1 would affect on glucose metabolism of the colorectal cancer cells. We found that cells knocked down by TMPO-AS1 produced less lactate (Fig. 3A). In addition, TMPO-AS1 knockdown inhibited ATP production and glucose uptake of SW480 cells (Fig. 3B, C). The data verified the repressing effect of TMPO-AS1 knockdown on the glucose metabolism of colorectal cancer cells.

### TMPO-AS1 binds with miR-1270 directly

**Fig. 4:**
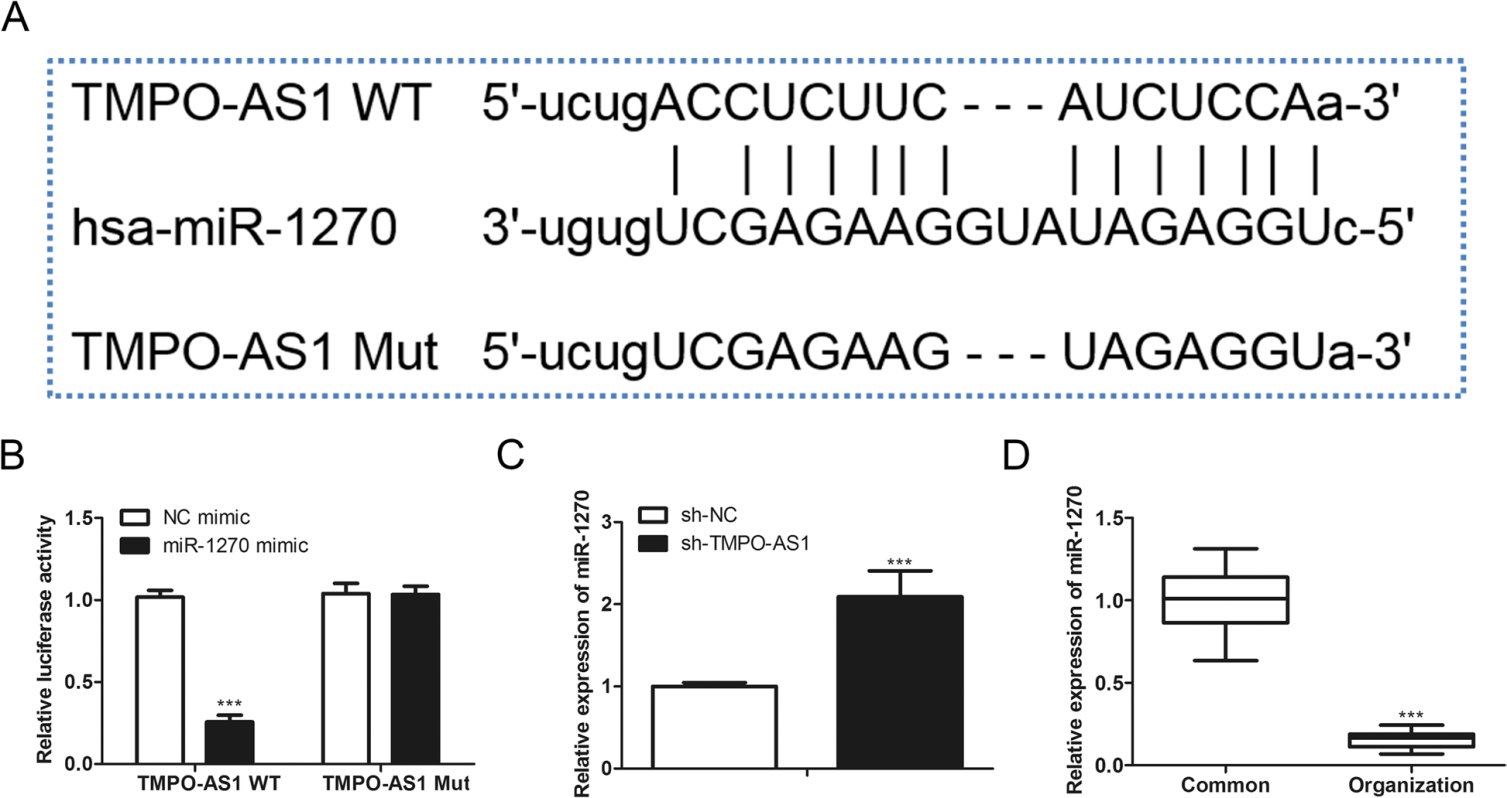
TMPO-AS1 serves as a miR-1270’s sponge. **A** The putative binding regions of TMPO-AS1 in miR-1270 were predicted through the Starbase database. **B** SW480 cells were performed transfection with a luciferase reporter vector combined with the miR-1270 mimics for 48 h, then the luciferase activity was determined. **C** MiR-1270 levels in the SW480 cells knocked down of TMPO-AS1 or not. **D** MiR-1270 levels in the tumor and in the normal tissues were assessed via qRT-PCR. *P < *0.05*, **P < *0.01*, ***P < *0.001*, *vs*. Control group.

LncRNAs often play their roles through sponging to miRNAs to work as the competitive endogenous RNA of miRNAs, leading to the inhibition of miRNA function. To elucidate the molecular mechanism of TMPO-AS1, we obtained the potential-binding miRNA of TMPO-AS1 through the starbase database, and it came to us that TMPO-AS1 possessed a potential-binding site with miR-1270 (Fig. 4A). Co-transfection of the wild type of TMPO-AS1 reporter plasmids with miR-1270 mimics induced an obvious decrease of luciferase activity in cells while showing no change in cells co-transfected with a mutant type of TMPO-AS1 reporter plasmids (Fig. 4B). TMPO-AS1 knockdown dramatically upregulated the expression of miR-1270 (Fig. 4C), indicating that miR-1270 can be bound to TMPO-AS1 directly. Besides, results from qRT-PCR displayed that miR-1270 was down-regulated in the colorectal tumor tissues (Fig. 4D). These statistics illustrated that TMPO-AS1 may function in oncogenic roles by serving as a sponge of miR-1270.

### Overexpression of miR-1270 suppresses the malignancy and glucose metabolism of colorectal tumor cells.

**Fig. 5:**
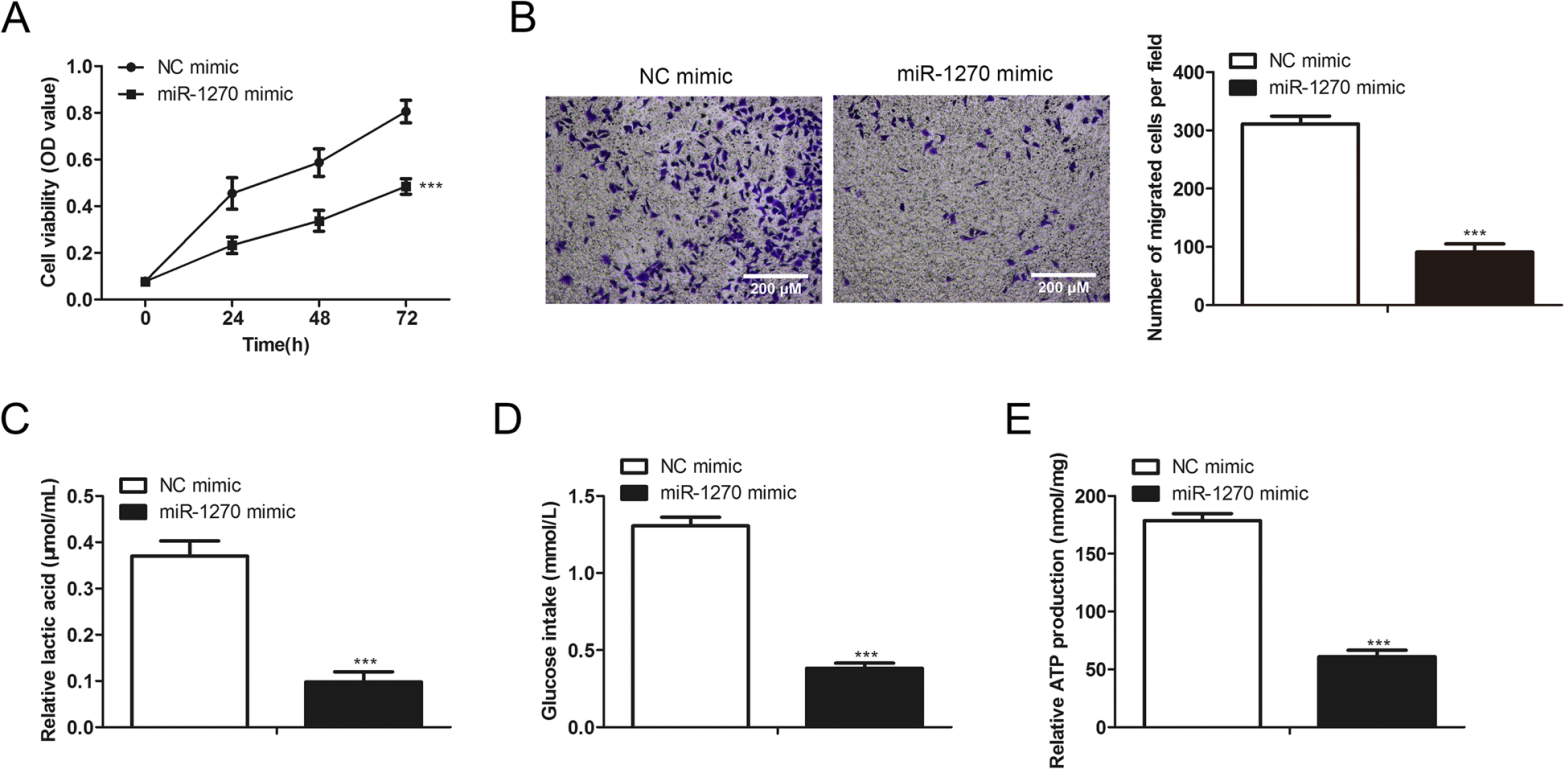
MiR-1270 overexpression suppresses the malignancy and glucose metabolism of colorectal tumor cells. The SW480 cells were transfected with miR-1270 mimics at the concentration of 50 pmol/mL and the relative negative controls for 48 h. **A** CCK-8 assay was performed to assess the cell viability and proliferation of SW480 cells. **B** SW480 cell's invasion ability was revealed through transwell assay. **C**-**E** The ATP production, lactate secretion, and glucose uptake of SW480 were verified by indicated commercialized assay. *P < *0.05*, **P < *0.01*, ***P < *0.001*, *vs*. Control group.

MiR-1270 influence on tumor cell invasion, proliferation, and glucose metabolism was explored to identify its function on colorectal cancer progression. We transfected miR-1270 mimics in the SW480 cells to overexpressed miR-1270. The results of cell viability exhibited that miR-1270 overexpression inhibited cell proliferation (Fig. 5A). Transwell assay revealed that miR-1270 mimics suppressed tumor cell invasive ability (Fig. 5B). Cells overexpressed with miR-1270 secreted less lactate (Fig. 5C). Overexpression of miR-1270 decreased the tumor cells' glucose uptake and ATP production (Fig. 5D, E). These findings demonstrated the suppressing role of miR-1270 on proliferation, invasion, and glucose metabolism of the colorectal tumor cells.

### MiR-1270 target PKM2 directly

**Fig. 6:**
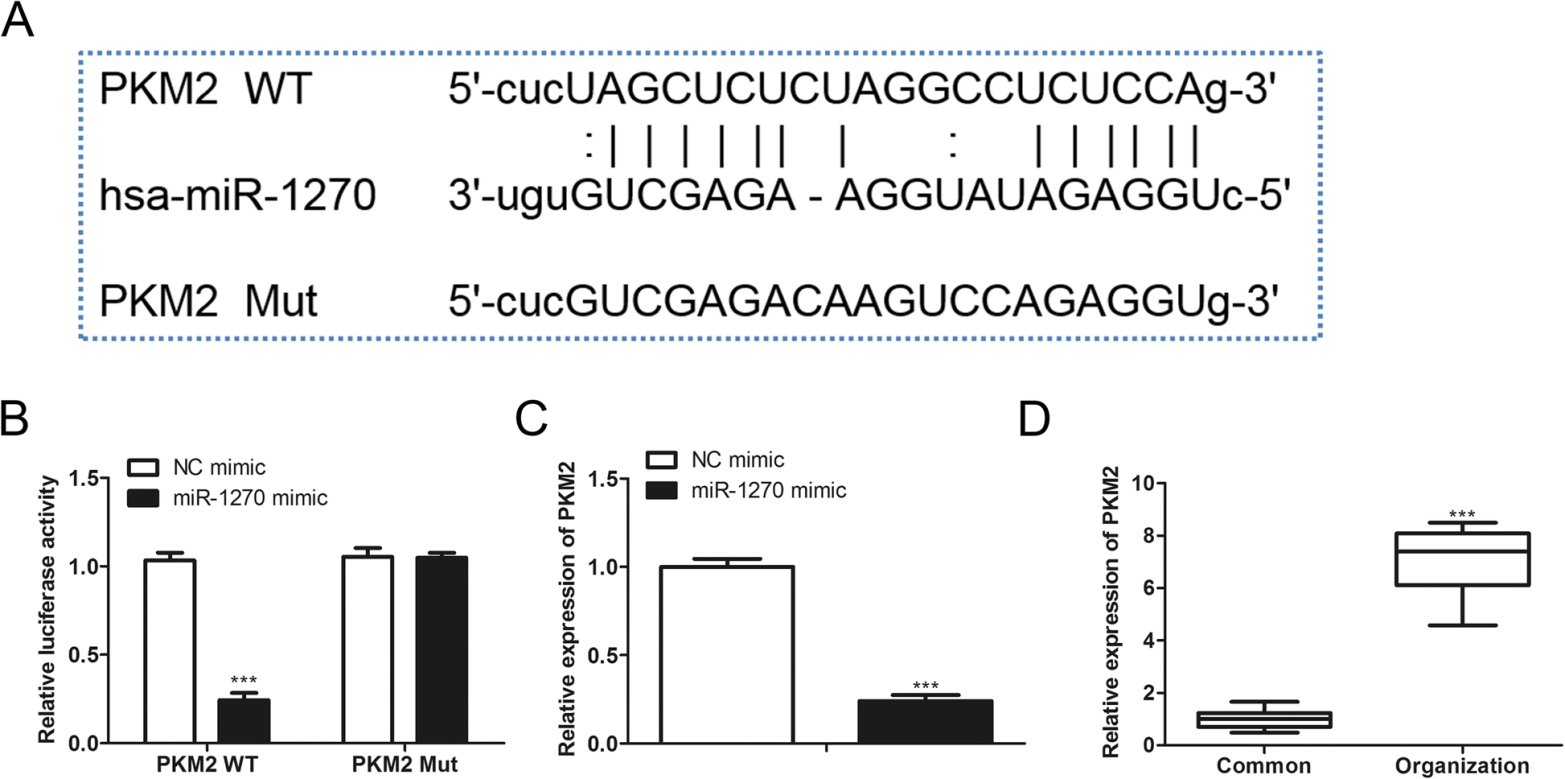
miR-1270 targets PKM2 directly **A** Starbase database displayed the supposing binding regions of miR-1270 in the seed region of 3'UTR of PKM2. **B** The SW480 cells were performed transfection with a luciferase reporter vector and the miR-1270 mimics for 48 h, following measurement of the luciferase signals. **C** The SW480 cells were transfected for 48 h with 50 pmol/mL miR-1270 mimics and relative negative controls, then the PKM2 expression was evaluated via qRT-PCR. **D** PKM2 level was detected in the tumor and in the normal tissues by qRT-PCR. *P < *0.05*, **P < *0.01*, ***P < *0.001*, *vs*. Control group.

Then we predicted the potential target of miR-1270 using the Starbase database and found that miR-1270 might be bound to the 3'UTR of PKM2 (Fig. 6A). Luciferase reporter assay revealed that co-transfection of the wild type of PKM2 reporter plasmids with miR-1270 induced an obvious decrease of luciferase signals in cells, while co-transfection of the mutant type of PKM2 reporter plasmids with miR-1270 mimics didn't influence the luciferase activity, indicating that miR-1270 was bound to the 3'UTR of PKM2 directly (Fig. 6B). Furthermore, transfection of miR-1270 inhibited PKM2 expression in SW480 cells (Fig. 6C). Besides, PKM2 was markedly upregulated in the colorectal tumor tissues (Fig. 6D). The above indicated that miR-1270 negatively modulated PKM2 expression through binding to the 3'UTR of PKM2.

### TMPO-AS1 regulates SW480 cell malignancy and glycolysis through miR-1270/PKM2 axis

**Fig. 7:**
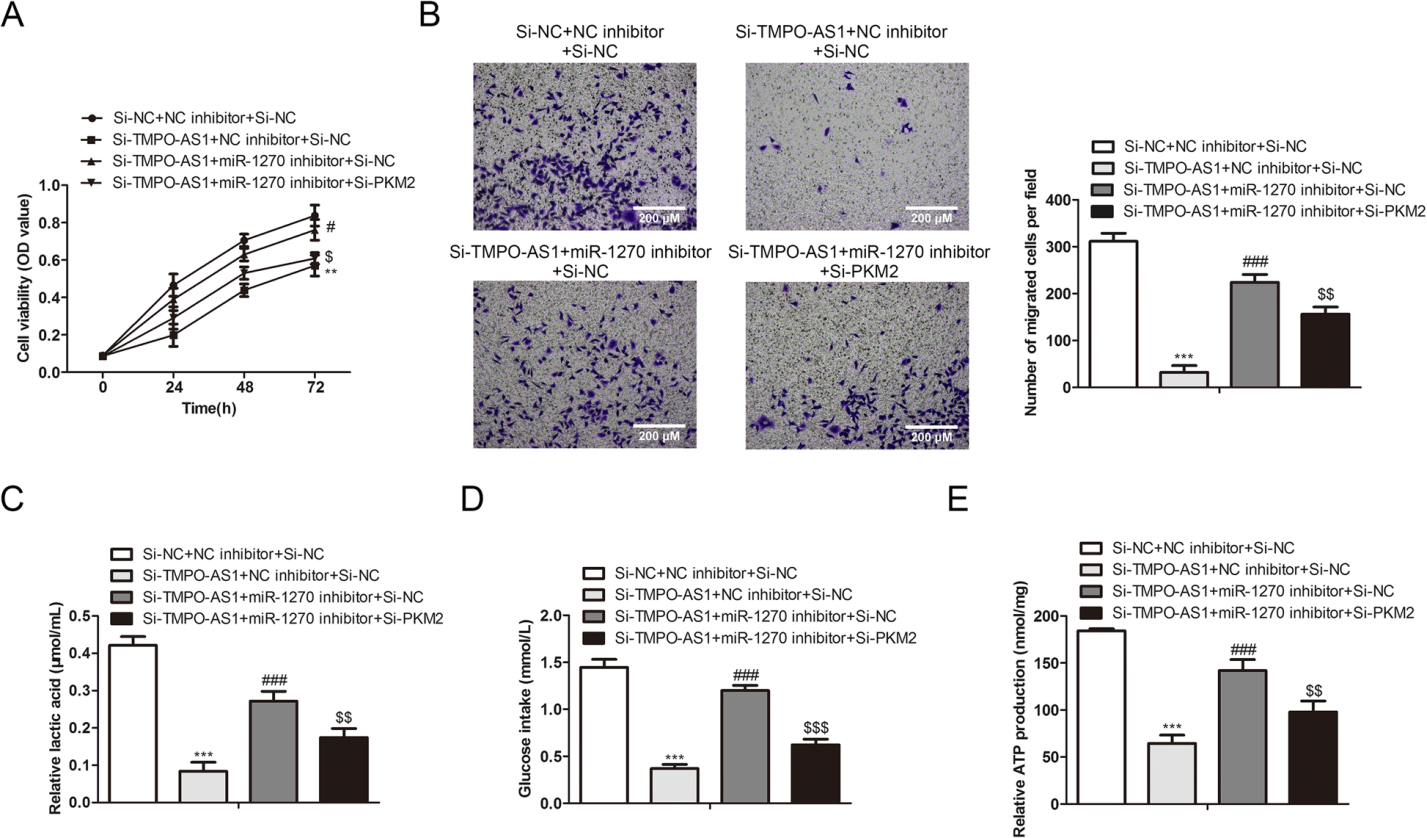
TMPO-AS1 regulates SW480 cell glucose metabolism and malignancy through miR-1270/PKM2 axis **A** SW480 cell viability was assessed via CCK-8 assay. **B** SW480 cells’ invasion ability was revealed through a transwell assay. **C**-**E** The lactate secretion, glucose uptake of SW480 and ATP production were gauged by indicated commercialized assay. *P < *0.05*, **P < *0.01*, ***P < *0.001*, *vs*. Control group.

Then we verified whether TMPO-AS1 regulated the cell glycolysis and malignancy through the miR-1270/PKM2 axis. First, TMPO-AS1 knockdown was discovered to inhibit invasion and proliferation in SW480, which were markedly reversed by transfection of miR-1270 inhibitors (Figs. 7A and B). Knockdown of PKM2 further revoked the miR-1270 effect on reversing the effect of TMPO-AS1 knockdown (Figs. 7A and B). MiR-1270 inhibitors altered the effect of si-TMPO-AS1 on inhibition of the lactate secretion, glucose uptake, and ATP production, while PKM2 knockdown further reversed miR-1270 inhibitors effect on the TMPO-AS1 knockdown (Figs. 7C-E). All these data implied that TMPO-AS1 modulated cell invasion, proliferation, and glucose metabolism through the miR-1270/PKM2 axis.

## Discussion

In recent years, lncRNAs have been found to participate in the metastasis, progression and programmed cell death of diverse cancers, including colorectal cancer [25, 26]. lncRNAs function as a clinical predictive marker of colorectal cancer through biological regulation of cell metastasis, proliferation, and apoptosis [27]. Besides, glucose metabolism’s role in colorectal cancer development has attracted researchers [28]. However, the studies of lncRNA in regulating glucose metabolism in colorectal tumors are still limited. This research clarified that TMPO-AS1 regulated glycolysis in colorectal cancer and exerted carcinogenesis via mediating miR-1270/PKM2 axis.

TMPO-AS1 draws much attention to its function in cancers, including breast cancer, bladder cancer, and pancreatic cancer. TMPO-AS1 was reported to upregulate the tumor tissues in colorectal cancer and correlated closely to metastasis [29]. Consistently, TMPO-AS1 was enriched in the colorectal tumor tissues, which predicted a poor prognosis for colorectal cancer patients in this research. Furthermore, TMPO-AS1 knockdown restrained proliferation, decreased the migrative and invasive abilities, and accelerated apoptosis in SW480 cells, following the previous reports of TMPO-AS1's oncogenic effect on other types of cancer cells [30, 31].

Glucose metabolism plays a vital role in tumor progression. Inhibition of glucose metabolism contributes to tumor cell death [23, 24], although some experimental evidence indicates that increased glucose metabolism may be related to stronger immune therapy resistance [32, 33]. Tumor cells require more aerobic glycolysis to produce ATP, with more lactate production and glucose uptake, called the Warburg effect [34]. In this study, the knockdown of TMPO-AS1 decreased ATP production, cell lactate secretion, and glucose uptake, indicating that TMPO-AS1 negatively regulated the glycolysis of the colorectal cancer cells. LncRNAs often played their roles through sponging to miRNAs to work as the competitive endogenous RNA of miRNAs, contributing to the inhibition of miRNA function. We confirmed that TMPO-AS1 was directly bound to miR-1270 by luciferase reporter assay and qRT-PCR. MiR-1270 is a tumor-suppressive miRNA [35, 36]. Accordingly, in this project, overexpression of miR-1270 suppressed the proliferative and invasive abilities and the glucose metabolism of tumor cells. Besides, we noticed that miR-1270 directly targeted to 3'UTR of PKM2 to downregulate the expression of PKM2. PKM2, coding M1 and M2 type of pyruvate kinase (PK), which participates in glycolysis, is the primary subtype of PK expressed in cancer cells [37]. PKM2 showed an oncogenic effect to promote cancer progression [38]. In this study, miR-1270 inhibitors abolished si-TMPO-AS1’s effect on suppressing the tumor cell proliferation, invasion, and glycolysis, while knockdown of PKM2 further reversed miR-1270 inhibitors effect on the TMPO-AS1 knockdown, indicating that TMPO-AS1 regulated tumor cell malignancy and glucose metabolism through miR-1270/PKM2 axis. We did not study the immune therapy resistance affected by the inhibition of glucose metabolism so further investigation is demanded to make clear the mechanism of tumor cell malignancy and glucose metabolism.

Moreover, Studies by Khanmi Kasomva et al. have shown that miRNAs, as primary regulators of mrna, can regulate the metabolic reprogramming of cancer cells either directly or indirectly by interacting with the glycolysis process during tumorigenesis [39, 40]. It has been found that mirnas can not only regulate glucose transporters (GLUTs/SLC2A), which is a key protein that helps glucose enter cells and control glycolysis, but also miRNA targeting GLUT is believed to play a role in regulating GLUT expression and enhancing glycolysis in a variety of cancer types such as prostate cancer [41] and liver cancer [42, 43].

In addition, mirnas can regulate a large number of enzymes involved in glucose, fatty acids, and amino acids metabolism, which are easily reprogrammed in cancer cells to meet higher metabolic demands. For example, the key regulator of the Warburg effect [44, 45], hexokinase (HK)2, a glycolytic enzyme found to be overexpressed in tumors, has been shown to enhance glycolysis and alter glucose metabolism in various cancers such as liver cancer cells [46] and breast cancer [47] by miRNA regulation [48]. In addition, glycolytic enzymes such as phosphofructokinase 1(PFK1) [49] and hepatic phosphofructokinase (PFKL) [50] are also regulated by miRNAs in cancer.

In a word, the present study provides a basis to clarify the relationship between TMPO-AS1 and the malignant degree of tumor cells and glucose metabolism.. Mechanically, TMPO-AS1 could increase PKM2 expression by playing as a competing endogenous RNA through binding to miR-1270 directly. This study provides a theoretical basis for TMPO-AS1/miR-1270/PKM2 regulatory network to become a new biomarker for the treatment of colorectal cancer.

### Supplementary Information

**Supplementary material 1.**

**Supplementary material 2.**

**Supplementary material 3.**

**Supplementary material 4.**

**Supplementary material 5.**

**Supplementary material 6. **

**Supplementary material 7.**

**Supplementary material 8.**

**Supplementary material 9.**

**Supplementary material 10.**

## Data Availability

The data used to support the findings of this study are included in the article.
