# Supplementary figures and images for: Long noncoding RNA TMPO-AS1 accelerates glycolysis by regulating the miR-1270/PKM2 axis in colorectal cancer
Source: BMC Cancer. 2024 Feb 21;24:238. doi: 10.1186/s12885-024-11964-w (PMC10880273; doi:10.1186/s12885-024-11964-w)

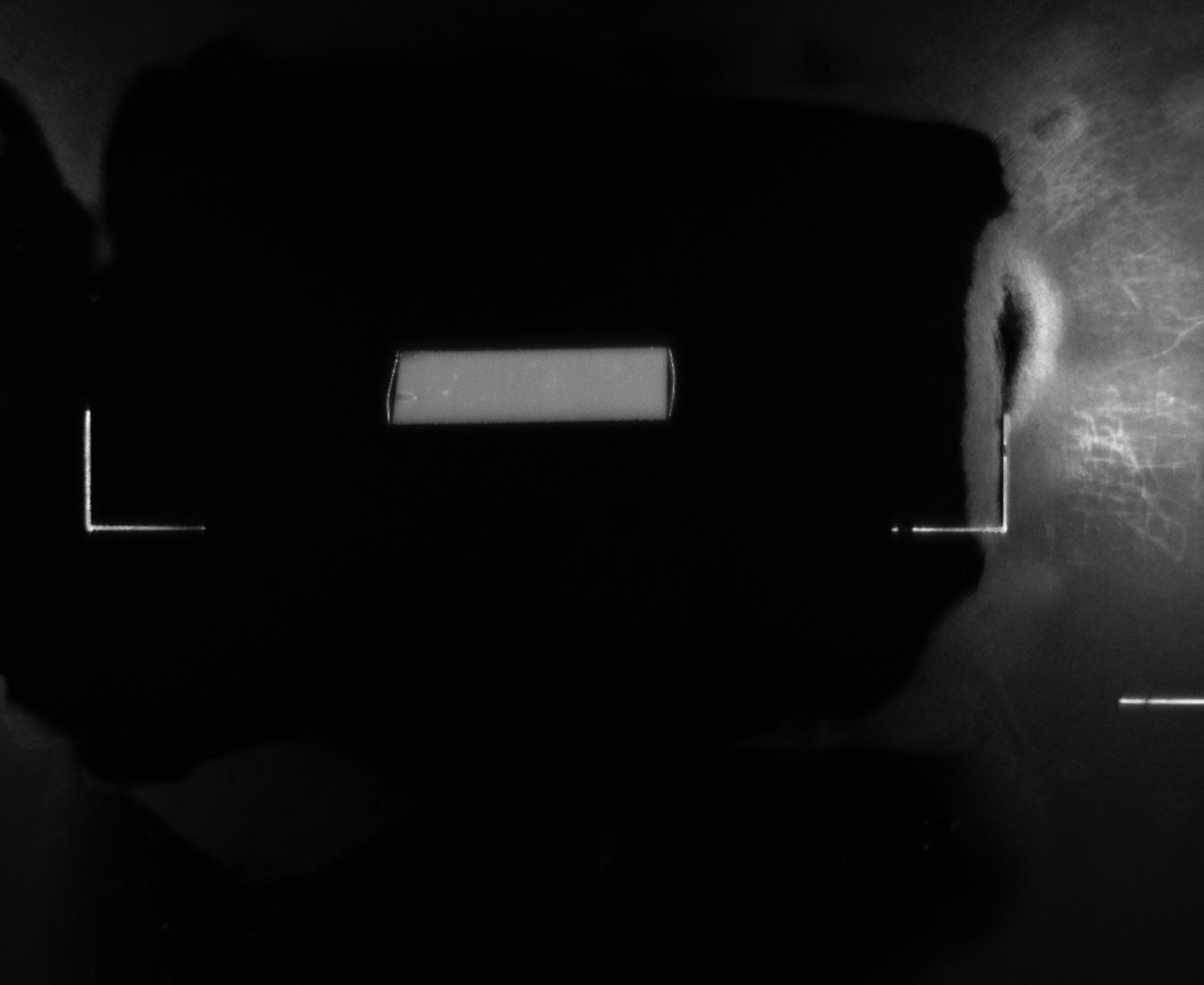

Supplement: Supplementary file 2 — Supplementary material 2. [file 12885_2024_11964_MOESM2_ESM.tif]

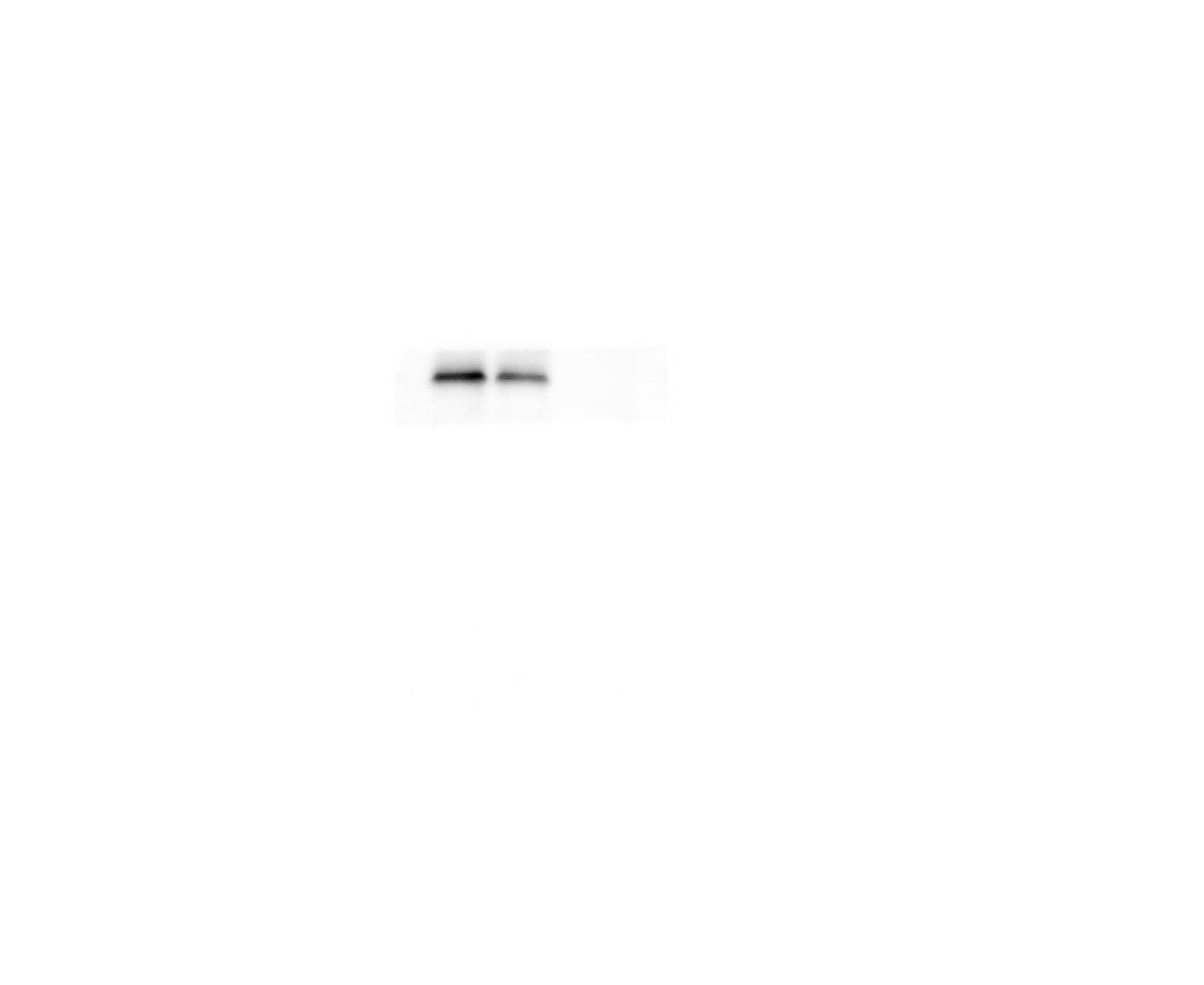

Supplement: Supplementary file 3 — Supplementary material 3. [file 12885_2024_11964_MOESM3_ESM.tif]

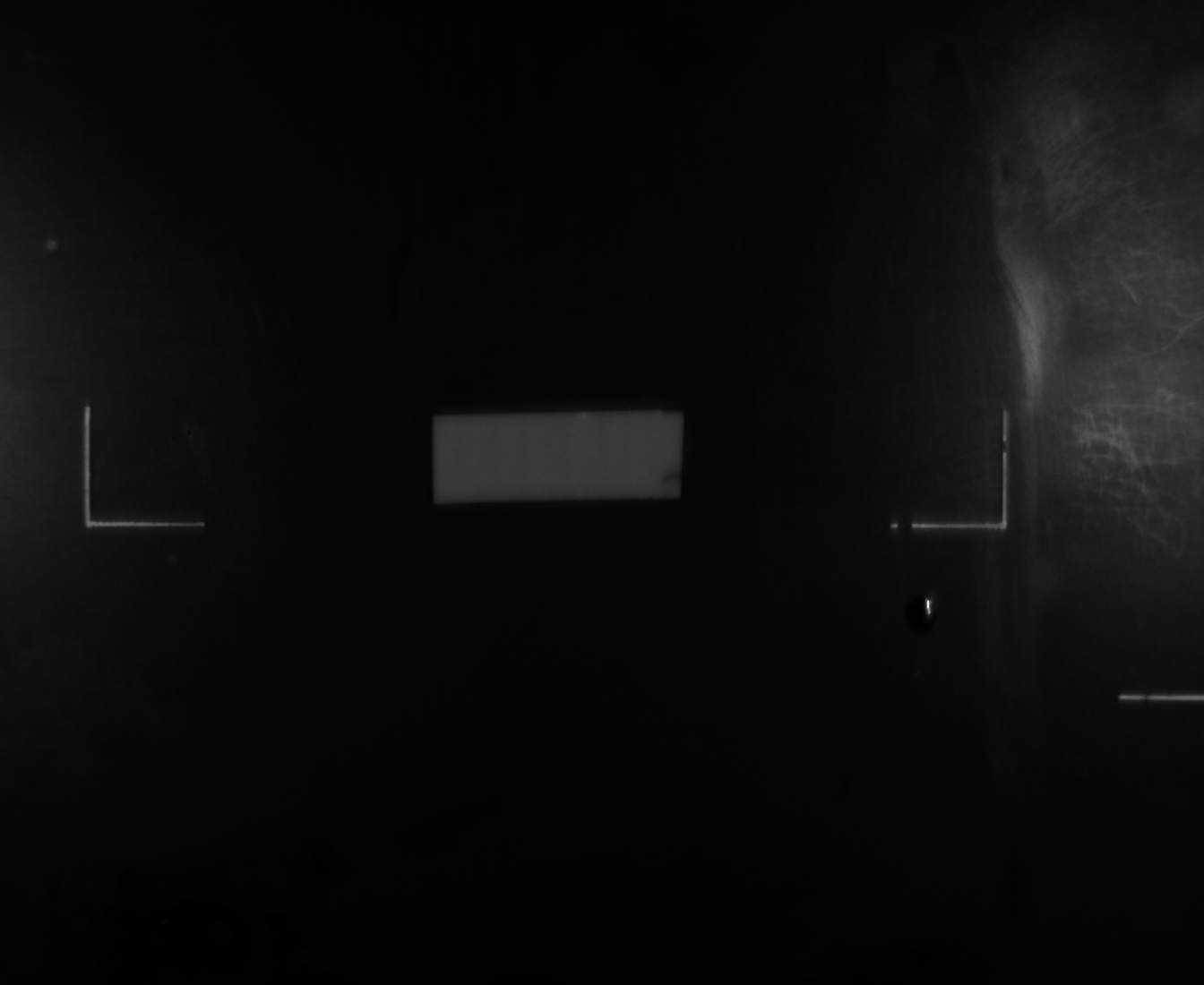

Supplement: Supplementary file 4 — Supplementary material 4. [file 12885_2024_11964_MOESM4_ESM.tif]

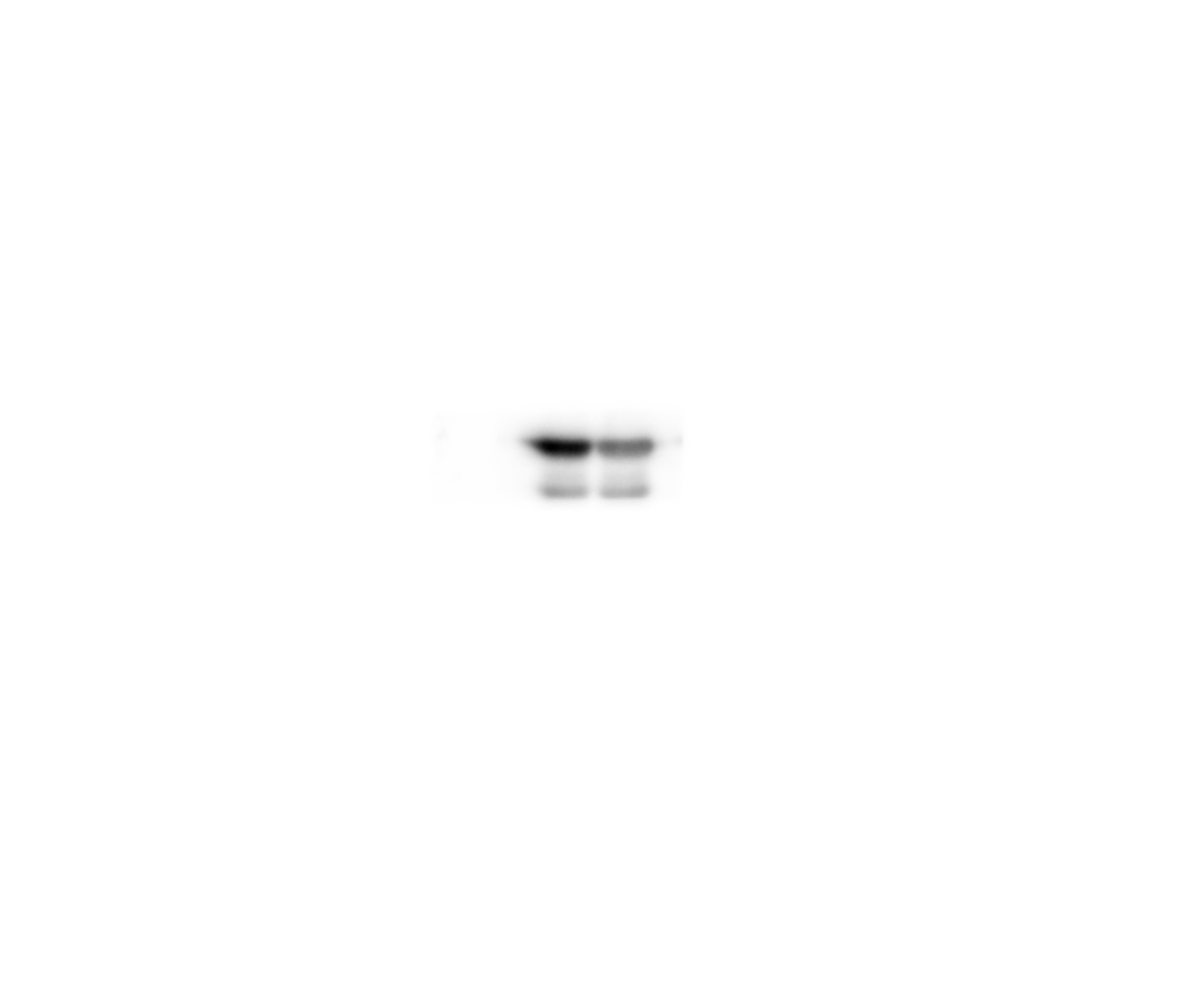

Supplement: Supplementary file 5 — Supplementary material 5. [file 12885_2024_11964_MOESM5_ESM.tif]

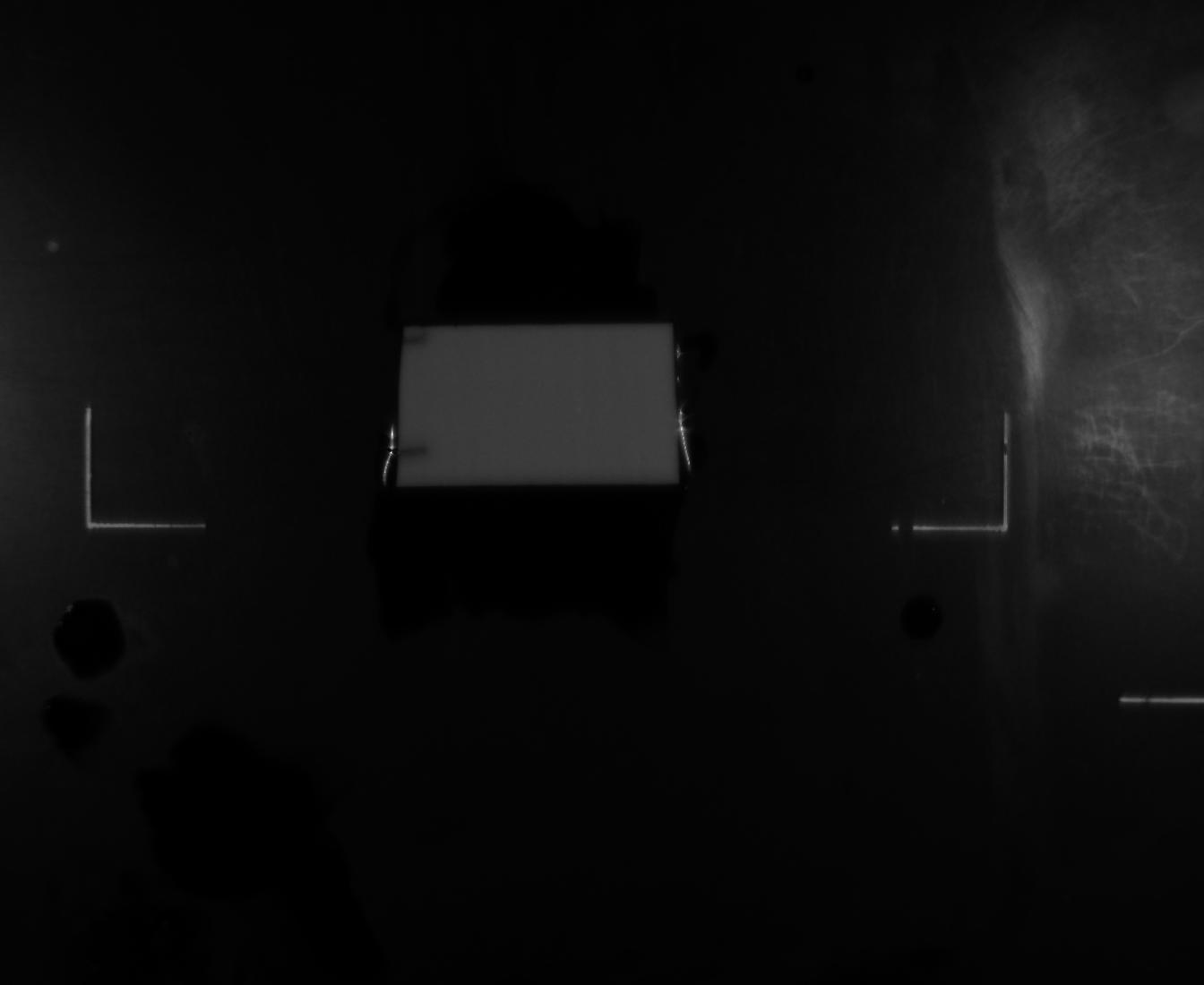

Supplement: Supplementary file 6 — Supplementary material 6. [file 12885_2024_11964_MOESM6_ESM.tif]

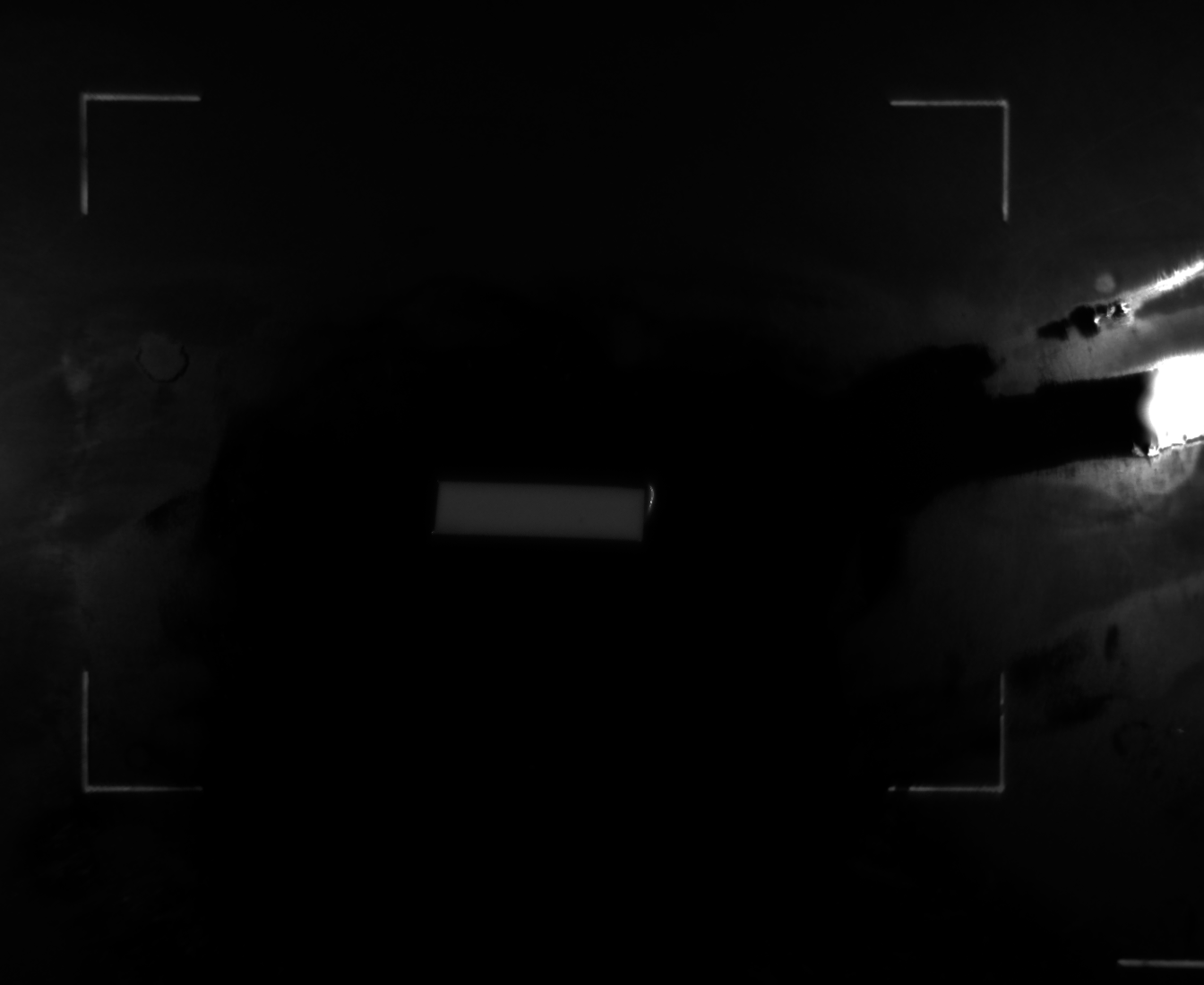

Supplement: Supplementary file 7 — Supplementary material 7. [file 12885_2024_11964_MOESM7_ESM.tif]

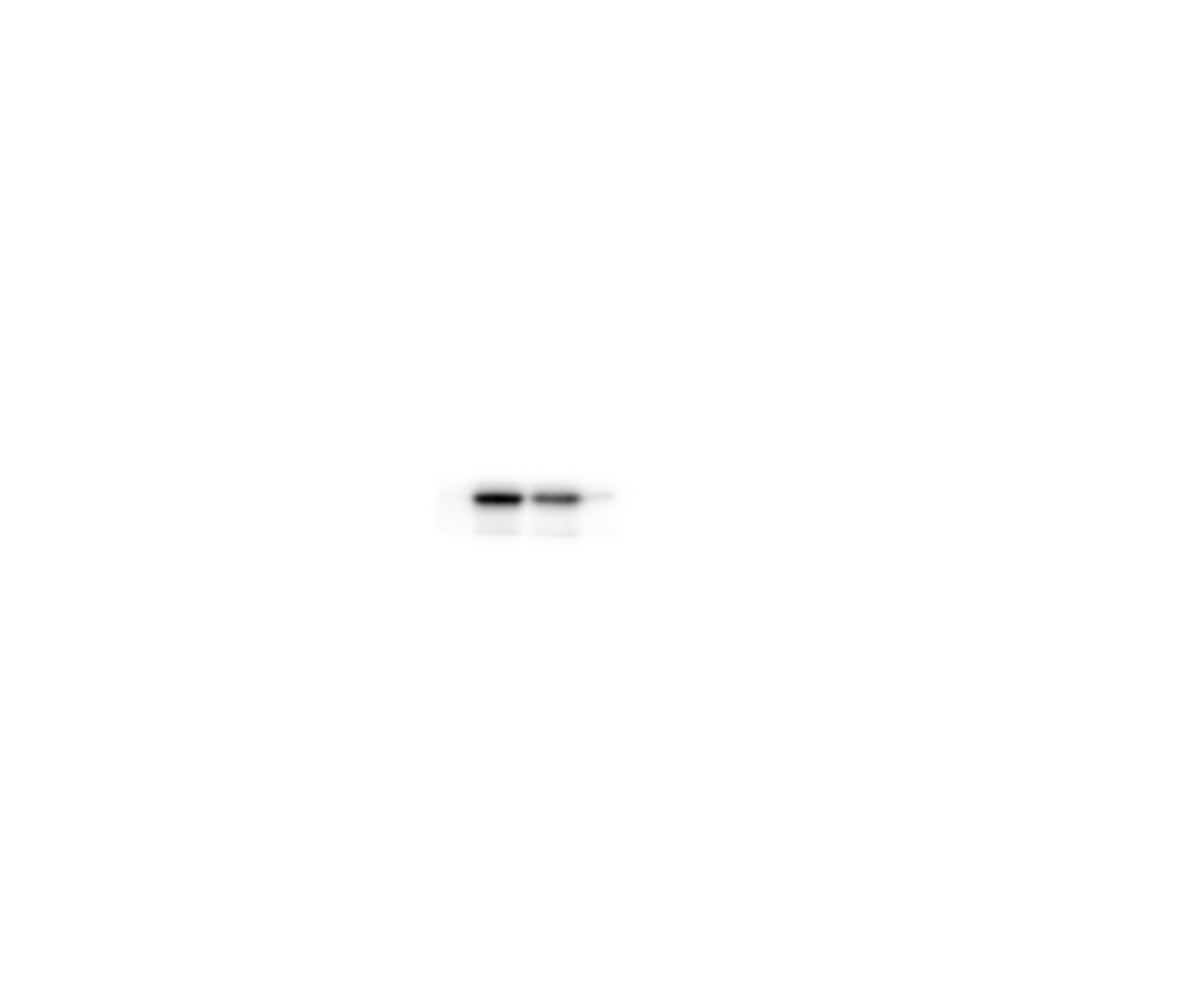

Supplement: Supplementary file 8 — Supplementary material 8. [file 12885_2024_11964_MOESM8_ESM.tif]

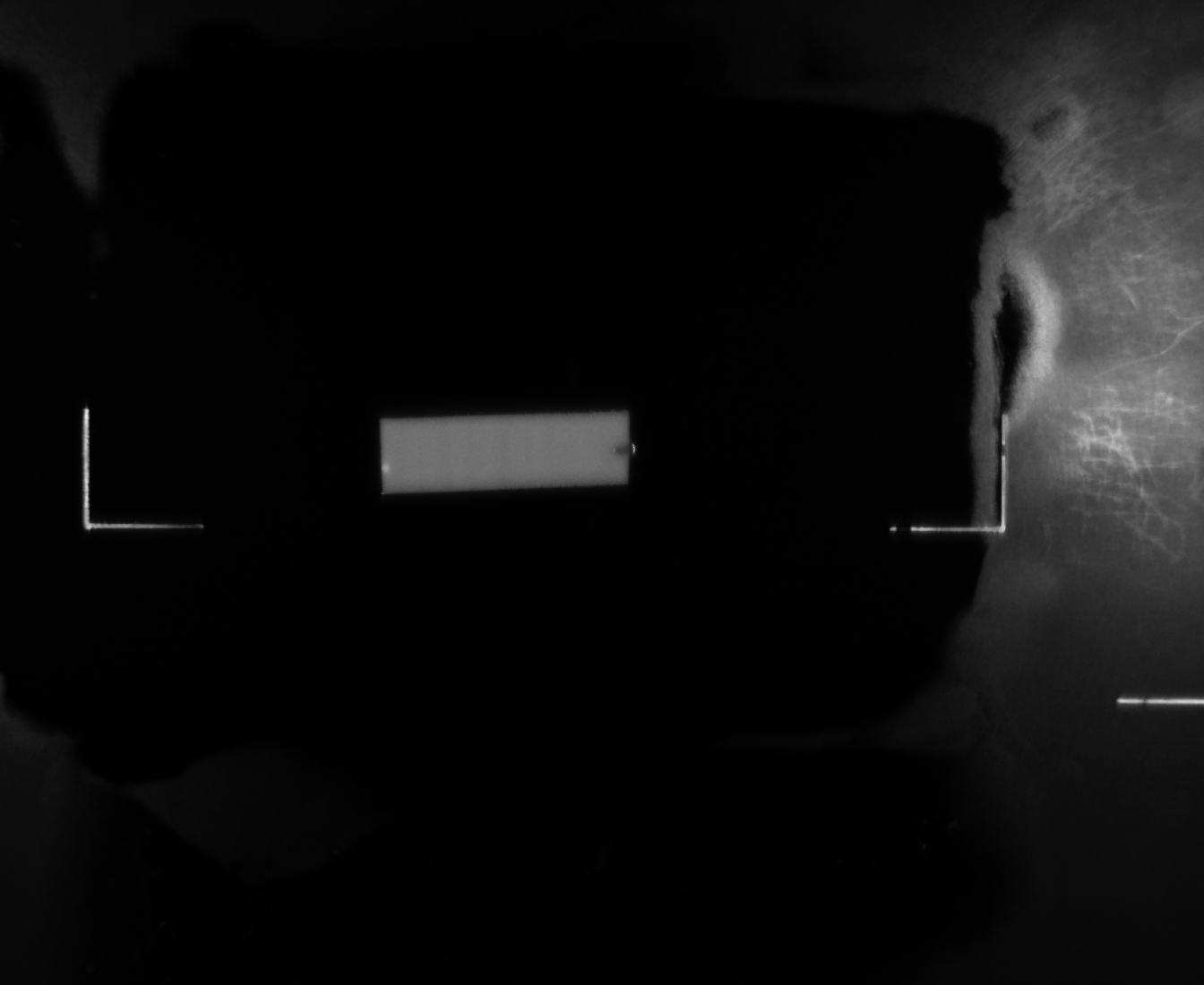

Supplement: Supplementary file 9 — Supplementary material 9. [file 12885_2024_11964_MOESM9_ESM.tif]

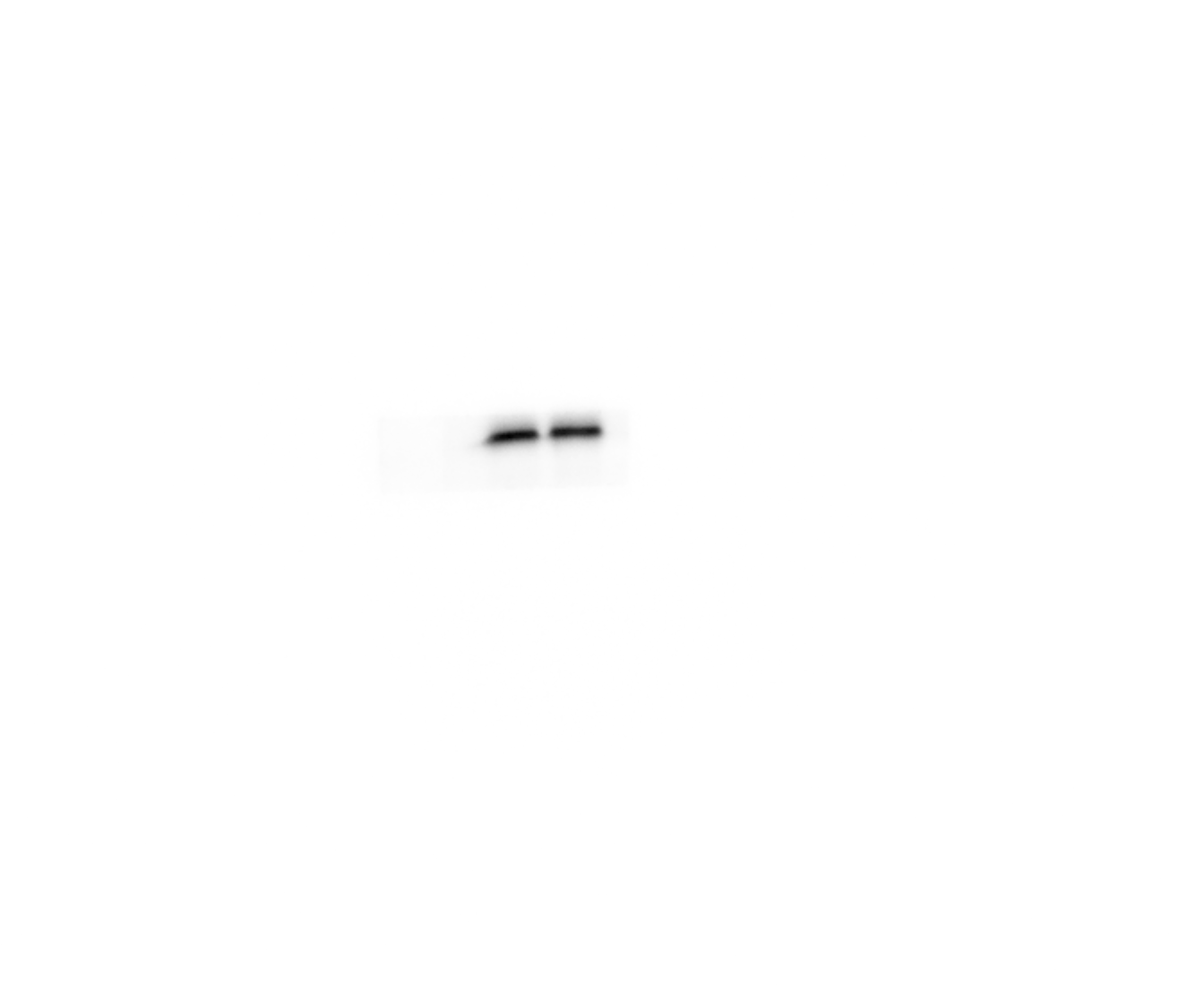

Supplement: Supplementary file 10 — Supplementary material 10. [file 12885_2024_11964_MOESM10_ESM.tif]
